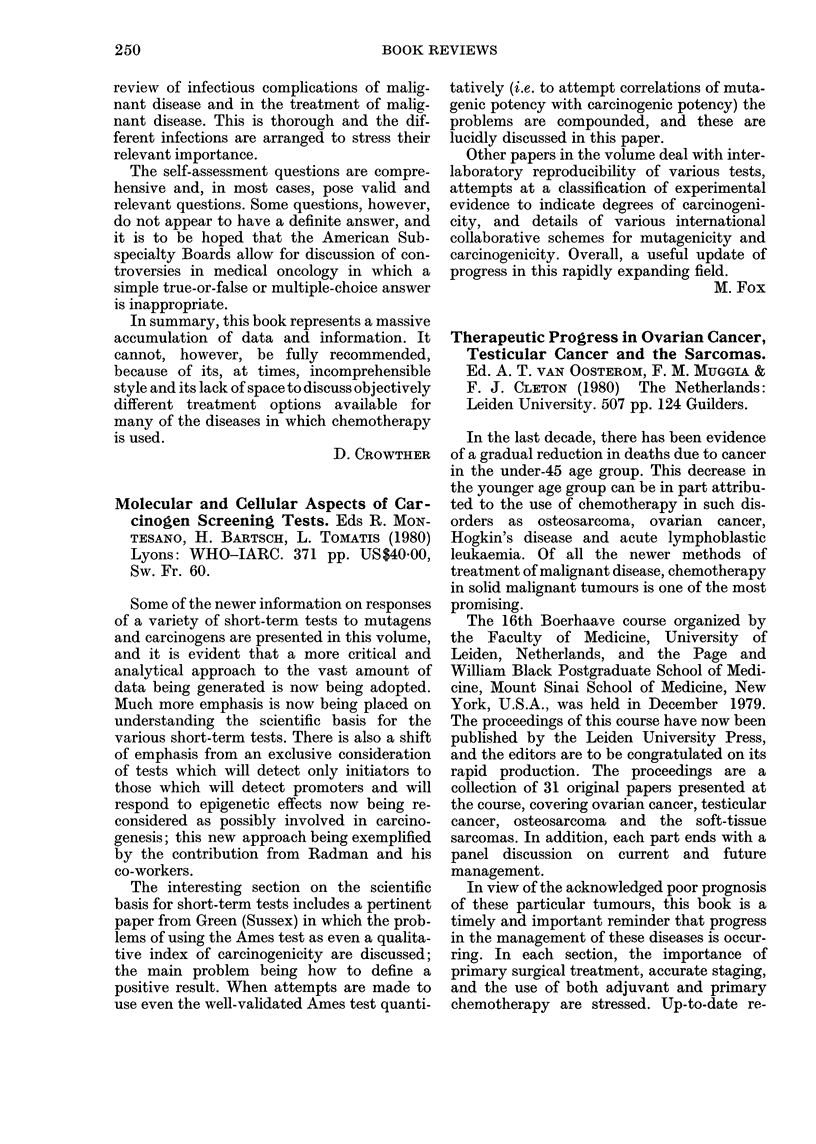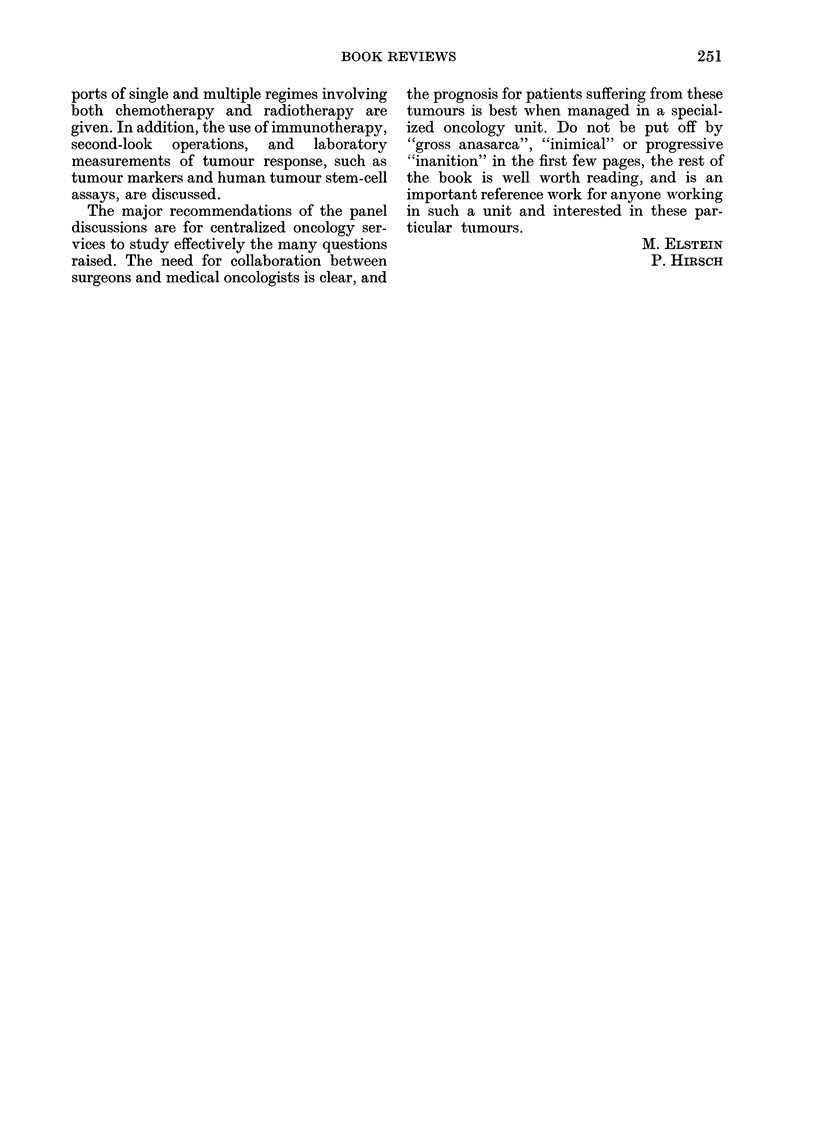# Therapeutic Progress in Ovarian Cancer, Testicular Cancer and the Sarcomas

**Published:** 1981-02

**Authors:** 


**Therapeutic Progress in Ovarian Cancer, Testicular Cancer and the Sarcomas.** Ed. A. T. VAN OOSTEROM, F. M. MUGGIA & F. J. CLETON (1980) The Netherlands: Leiden University. 507 pp. 124 Guilders.

In the last decade, there has been evidence of a gradual reduction in deaths due to cancer in the under-45 age group. This decrease in the younger age group can be in part attributed to the use of chemotherapy in such disorders as osteosarcoma, ovarian cancer, Hogkin's disease and acute lymphoblastic leukaemia. Of all the newer methods of treatment of malignant disease, chemotherapy in solid malignant tumours is one of the most promising.

The 16th Boerhaave course organized by the Faculty of Medicine, University of Leiden, Netherlands, and the Page and William Black Postgraduate School of Medicine, Mount Sinai School of Medicine, New York, U.S.A., was held in December 1979. The proceedings of this course have now been published by the Leiden University Press, and the editors are to be congratulated on its rapid production. The proceedings are a collection of 31 original papers presented at the course, covering ovarian cancer, testicular cancer, osteosarcoma and the soft-tissue sarcomas. In addition, each part ends with a panel discussion on current and future management.

In view of the acknowledged poor prognosis of these particular tumours, this book is a timely and important reminder that progress in the management of these diseases is occurring. In each section, the importance of primary surgical treatment, accurate staging, and the use of both adjuvant and primary chemotherapy are stressed. Up-to-date reports of single and multiple regimes involving both chemotherapy and radiotherapy are given. In addition, the use of immunotherapy, second-look operations, and laboratory measurements of tumour response, such as tumour markers and human tumour stem-cell assays, are discussed.

The major recommendations of the panel discussions are for centralized oncology services to study effectively the many questions raised. The need for collaboration between surgeons and medical oncologists is clear, and the prognosis for patients suffering from these tumours is best when managed in a specialized oncology unit. Do not be put off by "gross anasarca", "inimical" or progressive "inanition" in the first few pages, the rest of the book is well worth reading, and is an important reference work for anyone working in such a unit and interested in these particular tumours.

M. Elstein
P. Hirsch